# Identifying the Volcanic Eruption Depicted in a Neolithic Painting at Çatalhöyük, Central Anatolia, Turkey

**DOI:** 10.1371/journal.pone.0084711

**Published:** 2014-01-08

**Authors:** Axel K. Schmitt, Martin Danišík, Erkan Aydar, Erdal Şen, İnan Ulusoy, Oscar M. Lovera

**Affiliations:** 1 Department of Earth and Space Sciences, University of California Los Angeles, Los Angeles, California, United States of America; 2 Earth and Ocean Sciences, University of Waikato, Hamilton, New Zealand; 3 ATERRA R&D, Yuksel Cad. 30/8, Kizilay, Ankara, Turkey; 4 Department of Geological Engineering, Hacettepe University, Beytepe, Ankara, Turkey; University of Oxford, United Kingdom

## Abstract

A mural excavated at the Neolithic Çatalhöyük site (Central Anatolia, Turkey) has been interpreted as the oldest known map. Dating to ∼6600 BCE, it putatively depicts an explosive summit eruption of the Hasan Dağı twin-peaks volcano located ∼130 km northeast of Çatalhöyük, and a birds-eye view of a town plan in the foreground. This interpretation, however, has remained controversial not least because independent evidence for a contemporaneous explosive volcanic eruption of Hasan Dağı has been lacking. Here, we document the presence of andesitic pumice veneer on the summit of Hasan Dağı, which we dated using (U-Th)/He zircon geochronology. The (U-Th)/He zircon eruption age of 8.97±0.64 ka (or 6960±640 BCE; uncertainties 2σ) overlaps closely with ^14^C ages for cultural strata at Çatalhöyük, including level VII containing the “map” mural. A second pumice sample from a surficial deposit near the base of Hasan Dağı records an older explosive eruption at 28.9±1.5 ka. U-Th zircon crystallization ages in both samples range from near-eruption to secular equilibrium (>380 ka). Collectively, our results reveal protracted intrusive activity at Hasan Dağı punctuated by explosive venting, and provide the first radiometric ages for a Holocene explosive eruption which was most likely witnessed by humans in the area. Geologic and geochronologic lines of evidence thus support previous interpretations that residents of Çatalhöyük artistically represented an explosive eruption of Hasan Dağı volcano. The magmatic longevity recorded by quasi-continuous zircon crystallization coupled with new evidence for late-Pleistocene and Holocene explosive eruptions implicates Hasan Dağı as a potential volcanic hazard.

## Introduction

**Figure 1:**
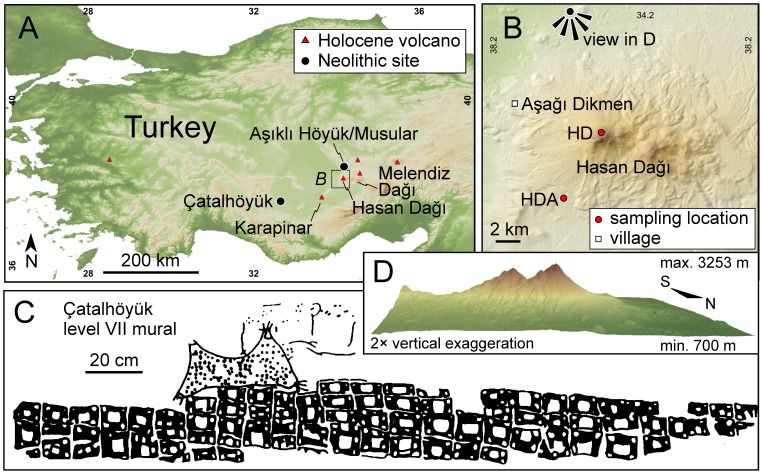
Location of the Çatalhöyük Neolithic site, Hasan Dağı, and other Holocene volcanoes in Anatolia. Overview map with inset showing map of sampling locations (A). Hasan Dağı volcano and sampling location of pumice dated in this study (B). Black-and-white rendering of Çatalhöyük wall painting (“shrine” 14; level VII) interpreted to show the twin-peaks of erupting Hasan Dağı and closely spaced buildings in the lower level [1]–[3] (C). An alternative interpretation is that of a leopard skin underlain by a geometric pattern [7]. Image reproduced from Fig. 2 in [7]. 3D rendering of Hasan Dağı twin peaks volcano as seen from N (D).

Starting from the discovery of the Neolithic settlement of Çatalhöyük in the early 1960s by British archaeologist James Mellaart, the excavations at this location have provided unique insights into the living conditions of humans at the transition from hunter-gatherer to settled agriculture societies. One outstanding find is a mural from level VII of Çatalhöyük (Fig. 1) famously described by its discoverer as depicting a volcanic eruption [1]–[3]. Similar interpretations, differing in detail, have been put forward since then, implicating this painting not only as the oldest depiction of a volcanic eruption, but as a contender for being the first graphical representation of a landscape or a map [4]–[6]. Detailed volcanological interpretations of the painting include reconstructions of the eruptive style with the summit region showing “falling volcanic ‘bombs’ or large semiliquid lava” [6]. According to these interpreters, the most likely candidate for the erupting volcano depicted in the upper register of the painting (Fig. 1) is the twin-peak volcano of Hasan Dağı, located ∼130 km NE of Çatalhöyük. This view, however, has been contested, largely because of the extraordinary age of the mural, and the absence of any other landscape art or map until much later in history [7] cf. [8]. The depiction of a leopard skin underlain by geometric patterns has been proposed instead [7].

A testable prediction of the volcanic eruption hypothesis for the Çatalhöyük mural is a geologic record of an eruption which would fall into, or briefly predate, the time when the Çatalhöyük mural was painted. Protracted periods of oral tradition over ∼250 generations have been proposed for prehistoric native North American myths following the ∼5700 BCE Mount Mazama eruption [5]. For the Çatalhöyük map (and volcano) hypothesis to be plausible, however, we surmise that a brief line of oral tradition, or even an eye witness portrayal, is perhaps more likely than tradition of a myth that detached itself from its inspiration in the physical world. This is not to say that realism must prevail in Neolithic art, but many of the apparent details can be reasonably expected to become lost or obscured during a long period of oral tradition. A tradition that predated the settlement of Çatalhöyük thus appears very unlikely, and hence we would predict a time period for the eruption between ∼7400 and 6600 BCE based on the ^14^C chronology of the Çatalhöyük cultural strata [9]. Neither proponents nor opponents of the “volcano” hypothesis for the Çatalhöyük painting have thus far scrutinized if and when such a volcanic eruption might have occurred.

## The Discovery and Semiotic History of the Çatalhöyük Mural

The Çatalhöyük “map” mural was first described by [2] as an approximately 3 m wide painting on the N and E wall of “shrine” 14 of excavation level VII (6430–6790 BCE; [9]). Originally identified as cultic spaces, “shrines” are now viewed to represent domestic areas with more-or-less cultic or ritual significance [10]. Upon excavation, the wall-painting was photographed in-situ [2], and subsequently publicized as a graphical reconstruction [3]. The original has since then been removed from the excavation site and it is presently curated in the Museum of Anatolian Civilizations in Ankara (Turkey). A reproduction is on display in lieu of the original at the excavation location.

The lower register of the mural (Fig. 1) contains ∼80 square-shaped patterns tightly arranged like cells in a honeycomb, and its upper register depicts an object that its discoverers initially identified either as a rendering of a mountain with two peaks with the cell-like patterns representing a plan view of a village with a general layout of the houses similar to that of Çatalhöyük and other nearby Neolithic settlements, or a leopard skin with its extremities cut off [1]–[3]. In the “map” interpretation, the volcano and its violent eruption are posited to have been significant for the inhabitants of Çatalhöyük because they procured obsidian in the vicinity of (albeit not directly from) Hasan Dağı [2]; cf. [11]. Alternatively, the natural spectacle of a cataclysmic eruption may have imprinted itself in the collective memory of the Çatalhöyük residents, charging the mountain with special cultic or religious significance [8]. In the “map” school of interpretation, different “villages” and “mountains” have been proposed by various authors, based on preferred topographic configurations that would provide the best match in shape and height of the twin-peak summits (with potential matches often assessed using landscape photography) with the corresponding fiduciary features in the painting. These scenarios include Hasan Dağı (a youthful volcanic edifice; [2], [12]), Melendiz Dag (a highly eroded volcanic complex; [13]), or Karapinar (a field of scoria cones; [5]) as the “mountain”, and Çatalhöyük [2] or Aşıklı Höyük [4] as the “village”. Whereas ^14^C ages for Asikli Höyük predate Çatalhöyük, the Aşıklı Höyük satellite site of Musular was coeval with the early to middle phase of Çatalhöyük [14]. Other archaeologists have dismissed the interpretation of a paired village-mountain altogether, and have reverted to Mellaart’s original “leopard skin” interpretation with a geometric pattern in the lower register [7]. This view is founded on the common and often central artistic representations of leopards in wall-paintings and sculptures recovered from Çatalhöyük, and the lack of any other archaeological records for maps in illiterate, non-urban societies [7]; cf. [4], [8].

## The Hasan Dağı Study Location

The Hasan Dağı (or Mount Hasan) stratovolcano has two characteristic peaks of similar elevation (3253 and 3069 m), forming Big and Small Mount Hasan. The composite edifice looms over the surrounding basins with their base elevation of nearly 1000 m. Its edifice was constructed over multiple stages identified as Paleo-, Meso-, and Neo-Hasan Dağı by extrusive dome emplacement and intermittent collapse events associated with ignimbrite volcanism [15]–[17]. Limited geochronological data [17] indicate emplacement of the oldest lavas at 7.21±0.01 Ma (K-Ar), and ignimbrites emplacement during an early caldera collapse at 6.31±0.20 Ma (^40^Ar/^39^Ar) which are contemporaneous with wide-spread Neogene ignimbrite volcanism in Cappadocia [18]. Only one K-Ar age for Meso-Hasan Dağı is published (∼0.58 Ma; [19]), and it is consistent with subsequent (<270 ka; [17], [19]–[20]) ignimbrite activity, dome extrusion with associated block and ash flow deposition, and peripheral scoria cones and maar eruptions that are collectively attributed to the Neo-Hasan Dağı stage. The Neo-Hasan Dağı edifice with its two summits is composed of collapsed andesitic to rhyodacitic lava domes creating a wide-spread apron of hot-emplacement pyroclastic deposits. The resulting nuée ardente deposits and interlayered lapilli-tephra beds are stacked in ∼10–20 m thick sequences which are exposed by channel erosion of the volcano’s flanks. Compositionally distinct rhyolitic lavas (including obsidian) and unwelded ignimbrites are restricted to the lower reaches of the Neo-volcanic edifice in the N, S, and W.

Available radiometric ages for Neo-Hasan Dağı dome lavas are from whole-rock or groundmass dating using K-Ar techniques [17], [19]–[23]. These ages indicate late Pleistocene activity, with an andesitic lava dome from the N flank of the volcano yielding a maximum age of 6 ka [16], and another andesitic lava flow erupted at the W base of the volcano (near Aşağı Dikmen village) with zero-age ^40^Ar [22]. Two summit domes yielded K-Ar ages of 29 and 33 ka [22]. These ages, while suggestive of very recent activity, lack independent confirmation, and in case of late Pleistocene K-Ar ages excess radiogenic ^40^Ar remains an untested possibility. No radiometric age determinations for pyroclastic deposits from Neo-Hasan Dağı were available prior to this study.

## Sampling

**Figure 2:**
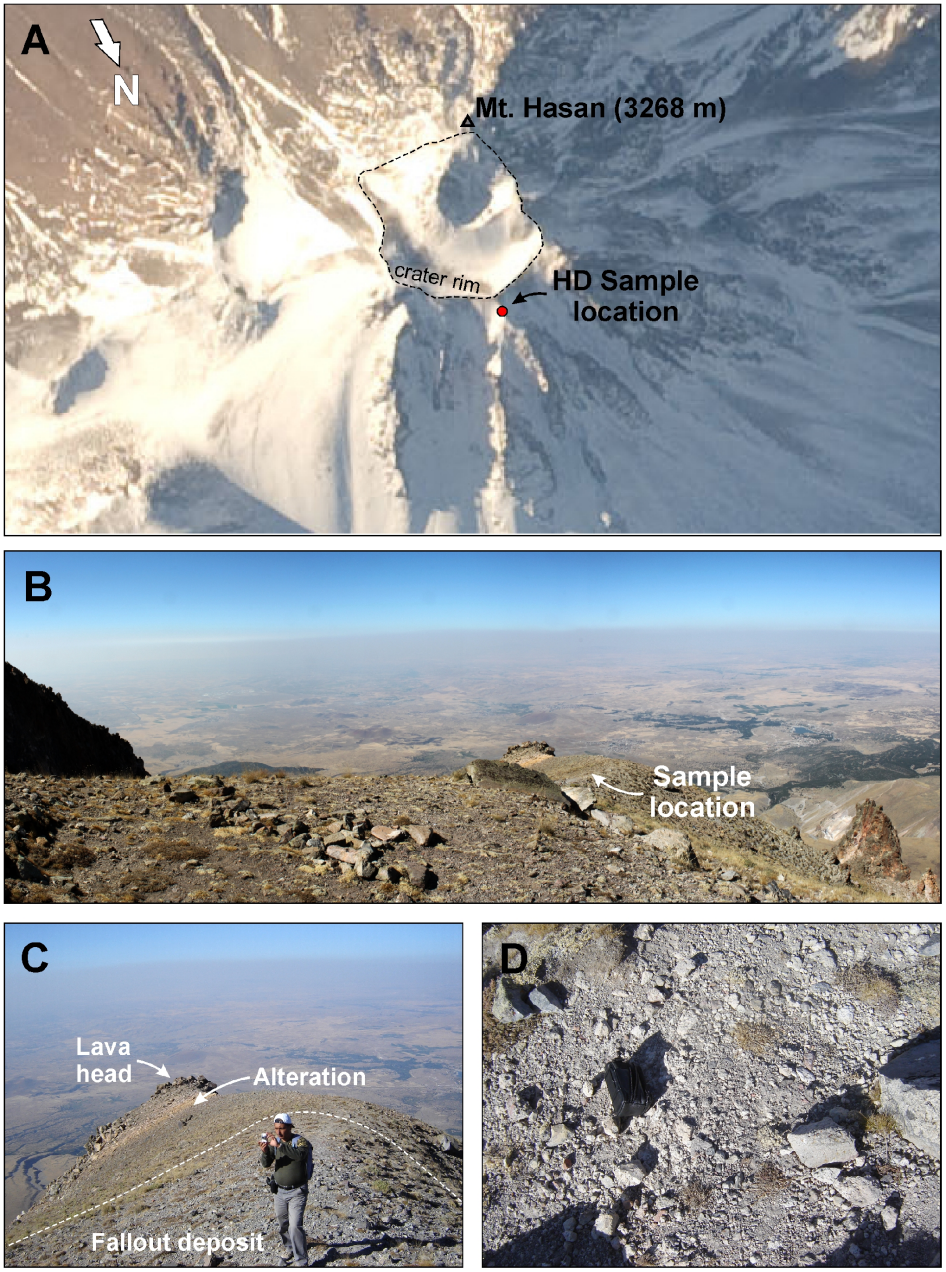
Location and field pictures for andesitic pumice deposit (sample HD) collected near the summit of Hasan Dağı. Astronaut photography of Hasan Dağı summit showing the location of sample HD (red dot) outside the crater rim (A). Image ISS022-E-5307 courtesy of the Image Science & Analysis Laboratory, NASA Johnson Space Center (http://eol.jsc.nasa.gov). Field scene of HD sampling location looking N (B). Light colored fall-out deposit abutting altered lava with geologist for scale (C). Close-up of pumice veneer at HD sampling location with camera pouch (center left) for scale (D).

Sampling complied with all relevant regulations, did not impact endangered or protected species, and did not require permits for the described study. Sample HD was collected from the summit region of Big Hasan Dağı peak (location 36S 602261E/4220954N; coordinates in UTM/WGS84 format). The outcrop is at 3160 m elevation, ∼22 m below the northern crater rim (Fig. 2 a). The deposit is located on a ridge with strewn pumice on the surface (Fig. 2 b-d). It is an unconsolidated single fall-out unit, lacking any major internal stratification except for potential reworking of the top 10–30 cm. Pumice clasts (9 cm maximum pumice diameter as average of the five largest clasts observed in outcrop) are angular, grey-white in color, with occasional pinkish discoloration. Lithic clasts comprise vitric lava and have an average maximum clast size of 7 cm. The second sample HDA is from the SW flank of the volcano (location 36S 599557E/4215676N) where pumice veneer was found as unconsolidated slope debris. A single pumice block ∼50 cm in diameter was collected. HD and HDA pumice as well as lithic clasts contain plagioclase and hornblende phenocrysts. Inductively coupled plasma (ICP) optical emission analysis revealed an andesitic composition of the sampled pumice clasts. Samples were retrieved from the subsurface by removing the top ∼20 cm of cover to exclude material possibly affected by reheating (e.g., lighting or wild fires).

## Analytical Methods

Zircon crystals were extracted from crushed and sieved rock powder. Matrix glass was dissolved through reaction with cold HF. The acid-insoluble mineral fraction was density-separated using heavy liquids (>3.3 g/cm^3^) to extract zircon. Large (>100 µm in width) euhedral crystals were then hand-picked and pressed into indium metal so that crystals’ prism faces were level with the mount surface. U-Th Secondary Ionization Mass Spectrometry (SIMS) analysis of crystal faces was conducted using established protocols for a CAMECA ims1270 in dynamic multi-collection mode [24]. Crater depths were ∼5 µm. A subset of crystals (preferentially those with old U-Th rim ages) was extracted for (U-Th)/He analysis using noble gas mass spectrometry (for ^4^He analysis) and ICP mass spectrometry (MS) for U and Th abundances following protocols in [25]. The remainder of crystals was subsequently grinded and polished to a depth of ∼20 µm to expose the interiors. The crystal interiors were then analyzed by SIMS in the same fashion as the rim analyses, permitting a direct comparison of rim and interior ages (between ∼20 and 25 µm depth) for the same crystals (Table 1). U-Th two-point isochron ages were calculated using SIMS zircon compositions and whole-rock U and Th abundances determined by ICP-MS (ACME Labs-Canada) as representative for the melt composition. Secular equilibrium was reasonably assumed for the melt given the overall longevity of the Hasan Dağı magma system, and an average value of (^238^U)/(^232^Th) = (^230^Th)/(^232^Th) = 0.882±0.015 was used as the model melt composition. The accuracy of U-Th and (U-Th)/He zircon ages was verified by analysis of secular equilibrium zircon standard AS3 (Duluth Gabbro) and FCT (Fish Canyon Tuff), respectively, interspersed with the unknowns. The resulting average values are: AS3 (^230^Th)/(^238^U) = 1.014±0.011 (2σ; mean square of weighted deviates MSWD = 0.63; n = 23) and FCT (U-Th)/He age = 28.0±0.87 Ma (MSWD = 0.06; n = 12).

**Table 1:** Summary of U-Th zircon ages.

Crystal name	Depth (µm)	(^238^U)/(^232^Th)	±1σ	(^230^Th)/(^232^Th)	±1σ	U (ppm)	Age (ka)	+1σ (ka)	−1σ (ka)
HD-10	0–5	4.403	0.071	1.752	0.146	274	30.7	6.2	−5.9
HD-11	0–5	4.762	0.083	3.244	0.314	169	102	26	−21
HD-12	0–5	4.439	0.030	1.760	0.157	260	30.6	6.6	−6.2
HD-13	0–5	5.054	0.057	1.638	0.293	304	21.6	9.8	−9.0
HD-1	0–5	3.865	0.064	1.755	0.096	535	37.4	5.2	−4.9
HD-2	0–5	4.363	0.064	1.557	0.091	369	23.2	3.6	−3.5
HD-3	0–5	6.622	0.114	2.114	0.197	226	26.2	4.9	−4.7
HD-4	0–5	6.130	0.062	2.071	0.238	161	27.8	6.6	−6.2
HD-5	0–5	4.527	0.150	3.241	0.165	250	113	18	−15
HD-6	0–5	5.176	0.068	3.056	0.207	319	76.8	11.4	−10.3
HD-7	0–5	4.069	0.093	4.007	0.151	435	431	∞	−148
HD-8	0–5	3.935	0.042	3.039	0.140	274	133	19	−16
HD-9	0–5	4.312	0.055	1.716	0.148	191	30.1	6.4	−6.1
HD-n1	0–5	4.393	0.042	1.388	0.140	253	16.7	5.2	−5.0
HD-n2	0–5	6.459	0.080	2.298	0.268	171	31.8	7.3	−6.8
HD-n3	0–5	7.030	0.112	2.541	0.275	173	34.2	6.9	−6.5
HD-n4	0–5	4.101	0.046	3.138	0.180	184	131	23	−19
HD-n5	0–5	5.905	0.045	1.835	0.234	136	22.7	6.5	−6.1
HD-n6	0–5	4.216	0.053	3.346	0.142	342	146	20	−17
HD-n7	0–5	6.254	0.086	5.473	0.343	139	210	65	−41
HD-n8	0–5	6.448	0.082	1.705	0.258	194	17.3	6.1	−5.8
HD-n9	0–5	4.005	0.056	1.800	0.212	176	37.6	11.1	−10.0
HD-n10	0–5	5.240	0.054	1.964	0.203	140	30.9	7.0	−6.6
HD-n11	0–5	3.878	0.039	3.565	0.193	208	246	108	−53
HD-n12	0–5	4.019	0.034	1.764	0.129	192	35.7	6.5	−6.1
HD-n13	0–5	3.935	0.022	2.304	0.184	187	68.1	13.1	−11.7
HD-n14	0–5	4.742	0.232	2.380	0.218	281	53.3	11.5	−10.4
HD-n15	0–5	4.773	0.053	2.945	0.139	328	82.2	8.8	−8.1
HD-n16	0–5	4.025	0.056	1.456	0.128	212	21.6	5.6	−5.3
HD-n17	0–5	3.434	0.096	1.634	0.153	187	37.7	9.9	−9.1
HD-n18	0–5	7.047	0.116	2.328	0.255	156	29.0	6.1	−5.8
HD-n19	0–5	6.636	0.067	3.497	0.379	276	66.0	14.1	−12.5
HD-n20	0–5	6.223	0.062	5.374	0.390	151	201	68	−42
HD-n1	20–25	4.146	0.021	2.742	0.254	85	91.8	21.8	−18.2
HD-n2	20–25	5.274	0.026	2.250	0.213	118	40.5	8.0	−7.4
HD-n4	20–25	2.253	0.010	2.493	0.169	78	∞	∞	∞
HD-n6	20–25	7.064	0.031	7.694	0.512	77	∞	∞	∞
HD-n8	20–25	4.987	0.024	1.657	0.255	84	22.6	8.7	−8.1
HD-n9	20–25	3.777	0.019	1.950	0.221	127	49.8	14.1	−12.5
HD-n12	20–25	4.284	0.030	2.046	0.193	70	45.4	9.9	−9.1
HD-n13	20–25	4.689	0.024	3.534	0.398	34	130	46	−32
HD-n14	20–25	4.258	0.022	4.230	0.220	176	521	∞	−237
HD-n15	20–25	4.813	0.060	4.751	0.456	116	453	∞	−233
HD-n16	20–25	6.068	0.027	3.354	0.410	63	70.5	17.9	−15.4
HD-n19	20–25	4.714	0.029	3.075	0.396	36	92.5	30.2	−23.6
HD-n20	20–25	5.843	0.029	6.364	0.502	102	∞	∞	∞
HDA-1	0–5	3.587	0.032	3.045	0.173	181	176	42	−30
HDA-10	0–5	3.584	0.044	2.102	0.152	300	66.0	11.9	−10.7
HDA-11	0–5	4.771	0.114	4.222	0.193	390	214	55	−36
HDA-12	0–5	6.045	0.030	2.520	0.180	362	41.9	5.7	−5.4
HDA-13	0–5	5.496	0.062	2.712	0.342	114	55.4	14.4	−12.7
HDA-14	0–5	3.734	0.021	3.477	0.110	475	263	62	−39
HDA-15	0–5	3.950	0.035	3.053	0.187	152	135	26	−21
HDA-16	0–5	3.814	0.044	1.898	0.141	183	46.8	8.4	−7.8
HDA-17	0–5	5.663	0.087	2.260	0.385	89	37.4	13.1	−11.7
HDA-18	0–5	4.343	0.085	3.611	0.228	195	170	43	−31
HDA-2	0–5	4.329	0.028	2.228	0.195	147	54.4	10.7	−9.7
HDA-3	0–5	4.720	0.154	2.040	0.158	161	39.5	6.9	−6.5
HDA-4	0–5	3.616	0.027	1.861	0.136	291	48.8	8.8	−8.2
HDA-5	0–5	4.505	0.063	2.790	0.132	306	82.0	9.0	−8.3
HDA-6	0–5	3.423	0.026	2.659	0.173	176	132	28	−22
HDA-7	0–5	5.790	0.029	3.132	0.254	159	67.2	11.0	−10.0
HDA-8	0–5	4.046	0.040	1.986	0.154	186	47.2	8.5	−7.9
HDA-9	0–5	4.165	0.056	2.092	0.182	172	50.6	10.1	−9.3
HDA-n1	0–5	6.349	0.049	3.119	0.261	176	57.7	9.2	−8.5
HDA-n2	0–5	8.424	0.101	4.913	0.510	90	83.7	17.2	−14.9
HDA-n3	0–5	4.610	0.076	2.510	0.250	158	63.0	14.0	−12.4
HDA-n4	0–5	3.887	0.047	3.611	0.265	248	261	396	−74
HDA-n5	0–5	8.122	0.039	2.811	0.486	83	34.0	10.5	−9.6
HDA-n6	0–5	4.686	0.089	1.905	0.209	107	34.5	8.6	−8.0
HDA-n7	0–5	3.349	0.219	3.432	0.266	284	∞	∞	∞
HDA-n8	0–5	3.873	0.023	2.028	0.150	176	53.1	9.3	−8.5
HDA-n9	0–5	3.822	0.028	2.398	0.148	264	79.5	12.1	−10.9
HDA-n10	0–5	5.173	0.167	3.275	0.458	104	89.4	30.9	−24.0
HDA-n11	0–5	3.861	0.042	3.302	0.183	221	183	44	−31
HDA-n12	0–5	7.455	0.063	5.569	0.476	98	136	32	−25
HDA-n13	0–5	4.188	0.060	2.558	0.202	210	77.6	14.6	−12.9
HDA-n1	20–25	4.711	0.026	3.219	0.417	140	103	36	−27
HDA-n2	20–25	4.264	0.024	4.044	0.273	157	299	∞	−88
HDA-n3	20–25	4.067	0.118	3.694	0.413	167	235	∞	−83
HDA-n5	20–25	4.391	0.049	4.081	0.358	183	265	∞	−84
HDA-n6	20–25	3.833	0.021	3.761	0.315	135	406	∞	−184
HDA-n8	20–25	4.770	0.032	4.239	0.263	244	218	75	−44
HDA-n9	20–25	3.175	0.017	2.781	0.136	439	193	46	−33
HDA-n10	20–25	4.630	0.024	4.640	0.292	238	∞	∞	∞
HDA-n11	20–25	4.126	0.024	4.471	0.322	209	∞	∞	∞
HDA-n12	20–25	6.265	0.133	6.324	0.450	137	∞	∞	∞
HDA-n13	20–25	5.418	0.026	4.769	0.309	202	213	71	−43
HDA-n14	20–25	3.065	0.050	1.881	0.098	486	67.3	9.7	−8.9
HDA-n15	20–25	3.130	0.033	2.919	0.124	445	259	102	−52
HDA-n16	20–25	5.238	0.024	4.044	0.458	109	142	53	−35

all uncertainties 1σ; decay constants used: λ_230_∶9.1577×10^−6^ a^−1^; λ_232_∶4.9475·10^−11^ a^−1^; λ_238_∶1.55125·10^−10^ a^−1^; age = zircon-melt two point isochron age for melt = (^238^U)/(^232^Th) = (^230^Th)/(^232^Th) = 0.882±0.015; ∞ secular equilibrium; sampling locations: HD = 36S 602261E/4220954N; HDA = 36S 599557E/4215676N (UTM/WGS84).

For young (<380 ka) accessory minerals, U-series disequilibrium corrections are significant for accurate (U-Th)/He dating [26]. This is because a deficit in ^230^Th at the time of zircon crystallization translates into a deficit of ^4^He produced by radioactive decay relative to secular equilibrium. Other disequilibria in U-decay series (e.g., ^231^Pa, ^226^Ra) are of secondary importance. To enable a correction for ^230^Th deficits, U-Th ages were determined for all zircons used for (U-Th)/He dating. In order to preserve enough crystal volume for subsequent He analysis, only U-Th zircon rim ages (of unsectioned crystals) could be determined. The interior ages of the zircons thus remain unknown, but they must fall between the rim crystallization age and secular equilibrium. This uncertainty was propagated into our (U-Th)/He age correction using the MCHeCalc software developed at UCLA. Because crystals which have old (near secular equilibrium) rim ages also have the least uncertainty regarding the disequilibrium correction, these crystal were preferentially selected for (U-Th)/He analysis, and their ages bear more strongly for the error-weighted average age.

## Results and Discussion

### U-Th Zircon Crystallization Ages

A total of 91 secondary ionization mass spectrometry (SIMS) spot analyses on rims and interiors of zircons from HD and HDA were conducted (Table 1). Nearly 50% of the 27 analyzed HD and HDA zircon interiors are in secular equilibrium with (^230^Th)/(^238^U) overlapping unity within 1σ uncertainty, and are thus older than ∼380 ka. Only a small number of rim ages (2 out of 64) are in secular equilibrium, whereas most rims show significant ^230^Th deficits attesting to their young age.

**Figure 3:**
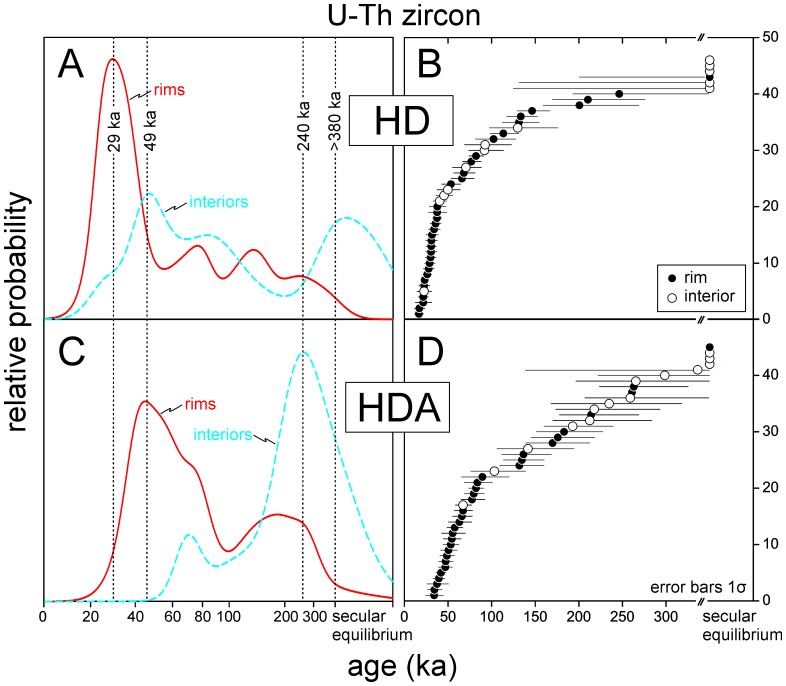
Relative probability and ranked order plots of U-Th zircon rim and interior crystallization ages for Hasan Dağı. Peak zircon crystallization ages for sample HD range between ∼29 ka and secular equilibrium (A, B). Peak zircon crystallization ages for HDA are between ∼49 ka and 240 ka, with some secular equilibrium ages present, mostly for crystal interiors (C, D). Error bars are plotted at 1σ for clarity.

The rim ages for HD and HDA overlap, but they show a significant difference in that HD zircon rim crystallization ages peak at ∼29 ka, whereas the youngest ages in HDA rims peak at ∼49 ka (Fig. 3). An ∼49 ka peak is also present in the interiors of the HD zircons which indicates that HD zircon nucleated on pre-existing zircon of HDA age, and continued to crystallize for several 10′s of ka. With few exceptions, interior ages are older than the corresponding rim ages on the same crystal. Only grain 8 of sample HD has indistinguishable rim and interior ages. This suggests that protracted zircon crystallization is typically recorded in individual crystals and the overall crystal population. No attempts were made to determine the age of secular equilibrium crystals or crystal domains through U-Pb analysis, but we speculate that the secular equilibrium crystals represent recycled crystal cargo from intrusive rocks dating back to the activity of Paleo- and Meso-Hasan Dağı.

This evidence for protracted zircon crystallization recorded in individual crystals and crystal populations adds to an increasing data body indicating that zircon longevity and recycling is common in long-lived volcanic systems in magmatic arcs such as the Cascades [27] or the Lesser Antilles [28]. In these cases, the origin of zircon has been ascribed to plutonic rocks which represent the unerupted residue of earlier magmatic pulses. Crystals from these plutonic rocks then became remobilized during subsequent stages of renewed magmatic activity. Such a scenario appears also plausible for Hasan Dağı: the presence of zircon (typical for evolved silicic melts) in comparatively primitive andesitic pumice suggests mixing of different magma types [16].

### (U-Th)/He Zircon Eruption Ages

**Figure 4:**
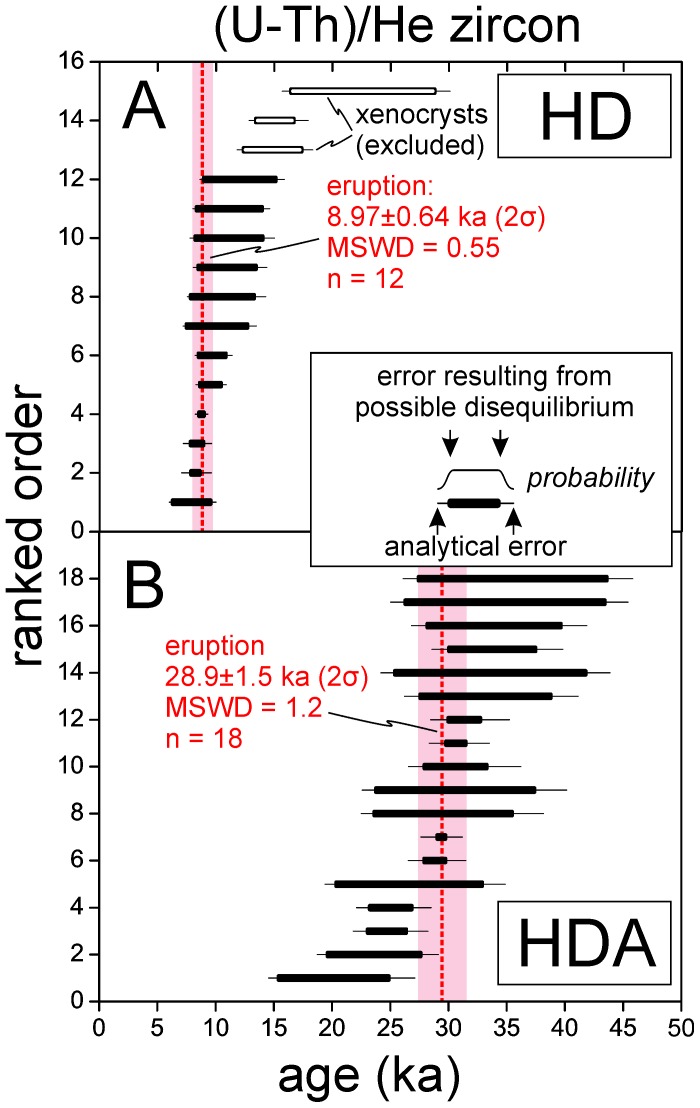
Ranked order plots for disequilibrium-corrected (U-Th)/He zircon ages for Hasan Dağı. Individual eruption ages (red dashed lines) for samples HD (A) and HDA (B) were calculated as error-weighted averages from (U-Th)/He zircon analyses. Errors comprise analytical uncertainties plus the uncertainty for the disequilibrium correction, and are bracketed by secular equilibrium (minimum age), and the disequilibrium-corrected age that corresponds to the measured U-Th rim crystallization age, assuming that it represents the crystallization age for the entire crystal (maximum age). Zircon crystals where rim ages are in (near-) secular equilibrium thus have the lowest uncertainties. Three crystals in sample HD yield (U-Th)/He ages that are too old to be reconciled with the average of the population. We interpret them as xenocrysts from pumice of older eruptions, and thus excluded them from the average. Weighted average age errors account for systematic and analytical uncertainties, and are quoted at 2σ; error bars plotted at 1σ.

Following the U-Th rim analyses, a subset of 15 and 18 crystals from HD and HDA, respectively, was selected for (U-Th)/He analysis (Fig. 4; Table 2). The selection was based on crystal size and integrity, with a preference for older rim ages because of the lesser impact of the disequilibrium correction (see below). In contrast to heterogeneous U-Th zircon crystallization ages in a long-lived magma system resulting from diffusive immobility of the ^238^U and ^230^Th parent-daughter pair, (U-Th)/He zircon ages for volcanic rocks are normally expected to uniformly record cooling upon eruption. We consequently calculated error-weighted averages for both samples which are 8.97±0.64 ka (n = 12) for HD and 28.9±1.5 ka for HDA (n = 18). Because the crystal interiors are inaccessible to direct isotopic analysis by SIMS unless a large portion of the crystal is removed to expose the interiors at the surface, we lack direct constraints for the interior ages. An equal probability for the crystallization age between the limits set by the rim age and secular equilibrium is assigned. The prevalence of secular equilibrium interiors is an indication that the younger ages (i.e., the left side of the thick error bar in Fig. 4) might be more likely, but we presently see no reliable way of how to assess this probability for individual crystals. The best strategy to minimize this uncertainty is therefore to target crystals with rim ages are at or close to secular equilibrium. These crystals, unfortunately rare in overall population, were preferentially analyzed for (U-Th)/He dating.

**Table 2:** Summary of U-Th and (U-Th)/He zircon ages.

Single crystal name	^232^Th (ng)	% ±	^238^U (ng)	% ±	4He (ncc)	% ±	TAU (%)	Th/U	F_t_	(U-Th)/He equilibrium age	±1σ	(U-Th)/He disequilibrium-corrected age	+1σ	−1σ
	(ng)		(ng)		(ncc)		(%)			(ka)	(ka)	(ka)	(ka)	(ka)
HD-1	0.686	1.438	0.949	1.835	0.000871	4.7	4.9	0.718	0.75	8.64	0.61	13.3	1.1	0.9
HD-2	1.38	1.43	1.78	1.84	0.00185	1.9	2.5	0.772	0.79	9.11	0.51	15.0	0.9	1.0
HD-3	1.03	1.43	1.49	1.84	0.00132	2.8	3.2	0.684	0.82	7.62	0.45	12.5	0.9	0.8
HD-4	2.54	1.43	3.42	1.83	0.00355	2.2	2.7	0.737	0.85	8.51	0.48	13.8	0.8	1.0
HD-5	3.26	1.43	5.14	1.83	0.00540	1.3	2.1	0.630	0.87	8.66	0.47	10.7	0.7	0.7
HD-6	1.67	1.43	2.54	1.84	0.00375	2.0	2.6	0.654	0.84	12.5	0.7	17.2	1.1	1.3
HD-7	1.97	1.43	1.99	1.84	0.00203	1.9	2.5	0.986	0.78	8.70	0.48	8.79	0.52	0.50
HD-8	1.05	1.43	1.27	1.84	0.00123	1.8	2.4	0.822	0.76	8.76	0.49	10.3	0.6	0.7
HD-9	0.853	1.436	0.825	1.835	0.000631	5.0	5.3	1.027	0.79	6.44	0.47	9.36	0.64	0.80
HD-10	1.79	1.43	2.96	1.84	0.00581	1.4	2.1	0.601	0.85	16.6	0.9	28.6	1.5	1.9
HD-11	0.967	1.435	1.090	1.835	0.001656	1.5	2.2	0.881	0.76	13.6	0.7	16.5	1.4	1.1
HD-12	0.934	1.436	1.488	2.225	0.00142	5.0	5.4	0.623	0.82	8.37	0.62	13.8	1.2	1.1
HD-13	0.696	1.438	0.992	1.855	0.000890	2.0	2.6	0.697	0.79	7.98	0.45	13.1	1.2	0.8
HD-n7	0.583	1.456	0.916	1.845	0.000774	8.4	8.6	0.633	0.76	7.98	0.79	8.76	0.87	1.02
HD-n11	0.322	1.466	0.439	1.858	0.000325	10.9	11.1	0.728	0.65	8.01	0.97	8.45	1.15	1.01
														
HDA-1	1.70	1.4	2.71	1.82	0.00735	2.8	3.2	0.625	0.84	23.2	1.4	26.2	2.0	2.0
HDA-2	1.74	1.4	1.60	1.83	0.00518	1.5	2.1	1.079	0.75	28.3	1.5	39.5	2.4	2.7
HDA-3	1.09	1.4	1.61	1.84	0.00474	1.2	2.0	0.674	0.82	25.6	1.4	41.6	2.3	2.3
HDA-5	2.01	1.4	2.94	1.83	0.00976	1.2	2.0	0.678	0.85	27.7	1.5	38.6	2.5	2.6
HDA-6	2.50	1.4	4.18	1.83	0.0151	1.2	2.0	0.593	0.86	30.2	1.6	37.3	2.5	3.2
HDA-8	0.944	1.4	1.33	1.84	0.00345	2.1	2.6	0.706	0.76	23.9	1.4	37.2	2.9	2.0
HDA-9	4.81	1.4	6.88	1.82	0.0237	1.2	2.0	0.695	0.88	27.6	1.5	43.4	2.4	3.2
HDA-10	2.63	1.4	4.23	1.83	0.0122	1.2	2.0	0.617	0.87	23.8	1.3	35.3	2.9	2.6
HDA-11	2.44	1.4	3.76	1.81	0.0133	1.8	2.4	0.645	0.84	30.1	1.7	32.6	2.7	2.0
HDA-12	1.14	1.4	1.57	1.85	0.00347	1.4	2.2	0.720	0.76	20.5	1.1	32.7	2.1	2.0
HDA-13	3.96	1.4	3.81	1.84	0.00963	1.2	1.9	1.032	0.84	19.8	1.1	27.5	1.7	2.2
HDA-14	2.28	1.4	3.18	1.84	0.0104	1.6	2.2	0.710	0.82	28.1	1.5	29.6	1.9	1.7
HDA-15	1.21	1.4	1.68	1.84	0.00561	1.5	2.2	0.719	0.84	28.1	1.5	33.1	3.1	1.7
HDA-16	0.927	1.4	1.47	1.84	0.00439	1.3	2.1	0.626	0.81	26.5	1.4	43.3	2.2	3.0
HDA-17	0.639	1.4	0.942	1.835	0.00171	4.1	4.4	0.674	0.83	15.6	1.0	24.7	2.4	1.9
HDA-18	1.95	1.4	2.75	1.81	0.00786	1.9	2.5	0.704	0.86	23.4	1.3	26.7	1.8	2.1
HDA-n4	2.05	1.4	2.85	1.84	0.00969	1.3	2.1	0.714	0.80	30.0	1.6	31.3	2.2	1.8
HDA-n7	4.80	1.4	2.84	1.85	0.0105	1.4	1.9	1.679	0.75	29.2	1.6	29.6	1.6	1.6

^4^ He (ncc) volume of helium in ncc at STP; Sm <0.01 ng; TAU = total analytical uncertainty; F_t_ = alpha retention factor; D_230_ calculated from zircon Th/U and HD Th/U = 3.4 and HDA Th/U = 3.5; sampling locations: HD = 36S 602261E/4220954N; HDA = 36S 599557E/4215676N (UTM/WGS84). Age averages are error weighted with the error defined by the equilibrium age minus analytical error, and the disequilibrium-corrected age plus analytical error.

Resetting of the (U-Th)/He ages by wildfires, lighting, or heating through lava can be excluded because the samples were collected from the subsurface, and away from any contacts with younger lava flows or domes. We therefore interpret these ages as dating the eruptive cooling of the HD and HDA pumice. Three HD crystals are, however, distinctly older than the remaining younger population. Because HD zircon was extracted from composite pumice, we suspect that an older pumice population was mixed into the deposit, either during the eruption or by post-eruptive reworking. The accuracy of the (U-Th)/He ages is underscored by their consistency with the U-Th crystallization ages, which always predate the eruption. The apparent gap in the youngest U-Th crystallization ages and the eruption is in agreement with the proposed magma mixing scenario whereby a crystalline mush or intrusion becomes remobilized by andesitic recharge. Rounding of the zircon tips is indicative of partial resorption of these crystals when they became entrained into the andesitic magma.

### Eruptive Ages Compared with Çatalhöyük Archaeological Ages

**Figure 5:**
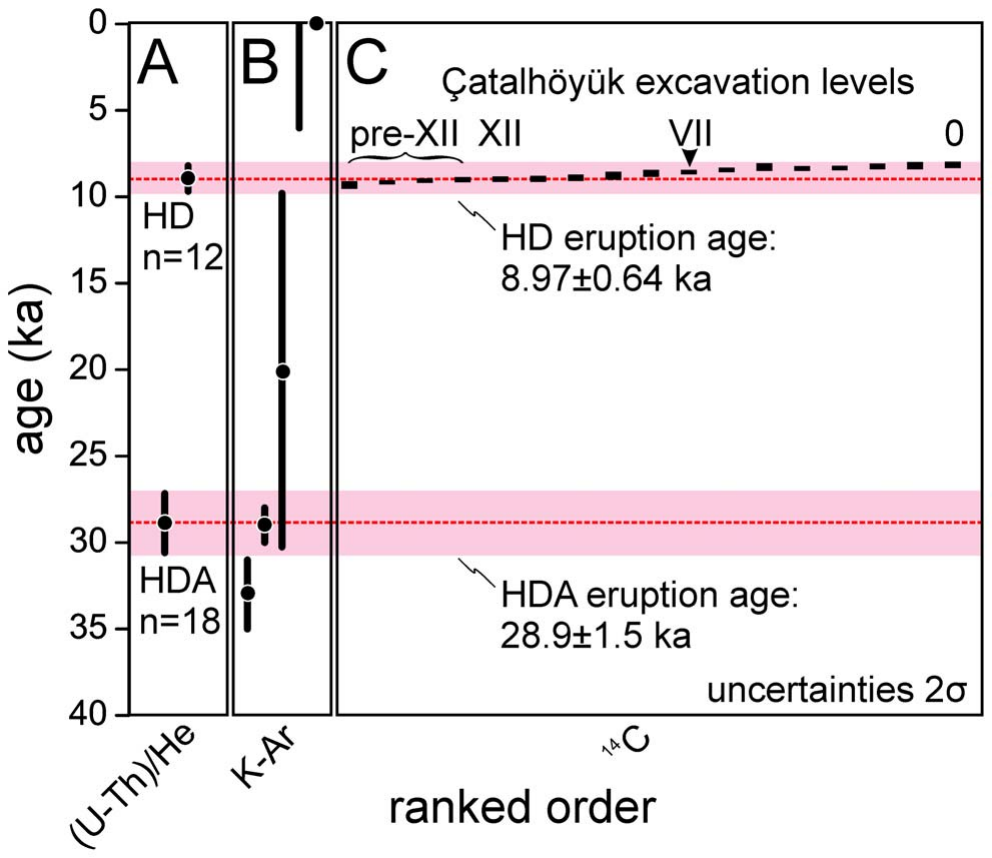
Comparison between Hasan Dağı (U-Th)/He zircon ages and published geologic and archaeological chronology. Ages for explosively erupted deposits based on (U-Th)/He zircon geochronology (A; this study). Published Hasan Dağı K-Ar ages of lava (B). ^14^C ages of cultural strata at Çatalhöyük including level VII containing the “map” mural (C). The HDA (U-Th)/He zircon age closely overlaps with a K-Ar age for a Hasan Dağı summit crater lava [23], and thus may represent the same eruptive episode. The HD (U-Th)/He zircon age of 8.97±0.64 ka (red bar projected over all panels) is the youngest (barring two K-Ar ages <0 ka and <6 ka age reported in [23] and [16], respectively), and best constrained Holocene eruption age for Hasan Dağı. It closely overlaps with Çatalhöyük ^14^C ages (level VII ∼8.4–8.6 ka; [9]). Pre-50 ka activity for Hasan Dağı is documented by K-Ar ages as old as 270±20 ka [20] which agree with the abundance of (near-)secular equilibrium U-Th zircon ages (Fig. 3).

Published K-Ar ages for lava samples from Hasan Dağı (Fig. 5) often have high uncertainties, or are maximum ages because of near-background ^40^Ar levels, and thus have little bearing on precisely dating Holocene eruptive activity at Hasan Dağı. There is, however, a late Pleistocene K-Ar age for a lava flow corresponding to the ∼29 ka (U-Th)/He age of sample HDA, suggesting contemporaneous effusive and explosive activity. New (U-Th)/He eruption ages for Holocene sample HD overlap closely with published ^14^C ages for the cultural strata excavated at Çatalhöyük (including level VII which contains the “volcano” wall-painting; Fig. 5). The eruption age for HD (6960±640 BCE in calendar years) is indistinguishable from the cultural occupation of Çatalhöyük within uncertainty, whereas published K-Ar ages (Fig. 5C) lack any overlap between the ^14^C ages for level VII. Analytical uncertainties of ^14^C and (U-Th)/He ages preclude any temporal correlation at less than millennial time scales, and therefore a residual uncertainty remains regarding the contemporaneity of the painting with the eruption. Nevertheless, our data are the first evidence for a volcanic eruption of Hasan Dağı coeval with human presence at Çatalhöyük.

The radiometric age, and the following geologic evidence corroborates the “volcano” hypothesis: (1) the HD deposit is the product of a low-elevation volcanic plume, presumably from a small-volume eruption, because it lacks wide-spread distal tephra; this is in accordance with the volcanological interpretation of the painting showing “mild Strombolian activity” [5]; (2) the deposit is present in the summit region of the taller peak (Big Hasan Dağı), as predicted from the painting; and (3) the hot emplacement of juvenile pumice (in contrast to a “cold” phreatic eruption) indicated by young (U-Th)/He ages implies magma ascent to shallow levels, and possibly an associated dome extrusion; this would represent a wide-visible eruption. Beyond the archaeological context, our results reveal recurrent explosive eruptive activity from a magma system that has been active for >380 ka. Additional mapping and dating is required to establish a more complete picture of how explosive eruptions of Hasan Dağı for the Holocene impacted the geologic, climatic, and anthropological evolution of the region.

## Conclusions

Combined U-Th and (U-Th)/He zircon geochronology provides the first radiometric age evidence for an explosive volcanic eruption of Hasan Dağı during the Holocene. The eruption age for pumice veneer from the summit of Hasan Dağı closely overlaps with the occupation of Çatalhöyük, and it therefore plausible that humans in the region witnessed this eruption. The geometric characteristics of the “volcano” in the upper register of the Çatalhöyük mural appear consistent with the location and fall-out deposition of the pumice. An older explosive eruption at ∼29 ka is evident from (U-Th)/He zircon dating of a pumice deposits at the base of Hasan Dağı. This age agrees with a K-Ar age for a lava flow from Hasan Dağı. The youngest zircon crystallization ages are within uncertainty of the eruption age, but some rim and interior ages predate the eruption by at least 380 ka. The volcanic edifice of Neo-Hasan Dağı is underlain by a long-lived magmatic system in which zircon quasi-continuously crystallized over several 100,000 years in an evolved silicic magma. Zircon-bearing magma from this reservoir was episodically remobilized and tapped in eruptions that involved thermal and compositional rejuvenation of the shallow silicic magma by more mafic injections from depth. In the light of the overall longevity of the Hasan Dağı magma system and radiometric evidence for Holocene eruptions, there is no indication that its activity is waning.
